# 基于出生队列的代谢组学分析新生儿出生体重与胎便代谢物之间的关系

**DOI:** 10.3724/SP.J.1123.2023.12012

**Published:** 2024-11-08

**Authors:** Yujie CHEN, Zhonghua LU, Shijia LIANG, Jie ZHANG

**Affiliations:** 厦门大学公共卫生学院, 福建 厦门 361102; School of Public Health, Xiamen University, Xiamen 361102, China

**Keywords:** 代谢组学, 出生队列, 液相色谱-高分辨质谱, 新生儿体重, 胎便, metabolomics, birth cohort, liquid chromatography-high resolution mass spectrometry (LC-HRMS), neonatal birth weight, meconium

## Abstract

新生儿出生体重是反映新生儿在宫内生长和发育情况的重要指标,其对儿童发育及成人健康状况评价具有重要的意义。胎便是新生儿在母体分娩后排出的最初粪便,被认为是研究母婴健康状况的理想生物样本。本研究依托孝感市妇幼保健院建立的出生队列,采用基于液相色谱-高分辨质谱的非靶向代谢组学技术,采集了484例新生儿胎便的代谢组数据,筛选出不同出生体重组间的差异代谢物,并探究了相关分子机理。在该出生队列中,低出生体重儿(<2500 g)和巨大儿(>4000 g)的发病率分别为3.3%和7.2%,与全国平均水平基本相当。正交偏最小二乘判别分析结果显示,与正常体重组相比,无论是低出生体重组还是巨大儿组,其胎便代谢组之间均存在显著差异(*P*<0.05)。实验还发现,低出生体重儿与正常体重儿之间以及巨大儿与正常体重儿之间的差异代谢物存在明显区别,分别指向不同的生物学途径。低出生体重儿胎便中的谷氨酸和脯氨酸等关键氨基酸的含量显著低于正常体重组(*P*<0.05),这可能与胎盘功能障碍和母体营养不足有关;与正常体重组相比,巨大儿胎便中的雌酮等激素代谢物水平显著升高(*P*<0.05),说明母体可能存在代谢性疾病或处于胎盘激素水平过高相关的生理状态。研究结果表明,胎便代谢物谱的差异可能是胎儿生长发育过程中不同代谢途径与调控机制的反映,该发现有望为胎儿发育相关疾病的深入研究提供潜在代谢标志物和研究方向。但是,本研究仅是基于孝感市出生队列开展,局限于特定的地区和人群,而建立多中心、多种族、多地区的研究方法将有助于验证研究结果的普适性。

## Abstract (en)

Neonatal birth weight is a crucial indicator of intrauterine growth and development with important implications for child development and adult health. The birth weight of a newborn is closely linked to the nutrition and health of the mother during pregnancy as well as genetic factors. Therefore, assessing the metabolic status of the fetus in utero is greatly significant for understanding the mechanisms responsible for abnormal birth weight. While previous studies often analyzed the impact of maternal metabolism on fetal development using umbilical cord blood from pregnant women, such blood may not accurately reflect the actual intrauterine environment owing to the barrier function of the placenta; moreover, obtaining biological samples during the fetal period is challenging. Meconium, the first feces excreted by a newborn, provides ideal biological material for studying maternal and infant health. Metabolomics can reveal metabolic changes in living organisms by analyzing small molecules in biological samples; hence studying meconium samples using metabolomics technology is expected to reveal fetal metabolic changes during pregnancy, thereby providing new insights into fetal nutritional intake, growth, and development, as well as metabolic pathways related to birth weight. To gain a deeper understanding of the metabolic changes associated with birth weight, this study collected metabolomic data from the meconium of 484 newborns in the established Xiaogan birth cohort using an untargeted metabolomics technique based on liquid chromatography-high resolution mass spectrometry (LC-HRMS) and analyzed the association between meconium metabolites and birth weight. This cohort exhibited incidence rates of low birth weight (<2500 g) and macrosomia (>4000 g) of 3.3% and 7.2%, respectively, which were roughly equivalent to the national average. Orthogonal partial least squares discriminant analysis revealed significant differences between the meconium metabolomes of the low birth weight and macrosomic groups when compared to the normal weight group. We discovered significant distinctions between the differential metabolites of newborns of low birth weight and those of normal weight, as well as between macrosomic and normal weight newborns that point to disparate biological pathways. Newborns with low birth weight exhibited significantly lower levels of critical amino acids, such as glutamate and proline, compared to the normal weight group, which may be associated with placental dysfunction and maternal nutritional deficiencies. Conversely, the meconium of macrosomic newborns contained significantly elevated levels of hormone metabolites such as estrone that reflected the pathophysiological state associated with maternal metabolic diseases or excessive placental hormone levels. Our study suggests that the metabolomic profile of the meconium reflects the metabolic pathways and regulatory mechanisms at play during fetal growth and development, and offers potential metabolic biomarkers and directions for future in-depth research into diseases related to fetal development. However, this study was based solely on the Xiaogan birth cohort, which was limited to specific regions and populations. A multicenter, multiethnic, and multiregional study is expected to help validate the universality of our research findings.

新生儿的健康问题已成为社会广泛关注的焦点,因为这不仅关系到个体的未来健康,也影响着国家的生产力和整体卫生水平。新生儿的出生体重是反映其在宫内生长和发育情况的重要指标,对新生儿的健康状况评估至关重要。低出生体重儿和巨大儿的发病率在不同地区和人群中存在差异,全球低出生体重儿的发生率为15.5%,主要集中在发展中国家,而我国低出生体重儿的发生率为4%~8%,仍然高于发达国家水平。随着我国经济的快速增长,巨大儿的发病率也不断上升,目前已经达到7%~8%,虽然我国巨大儿的发病率低于大多数欧美国家^[1,2]^,但却远高于日本^[3]^和西班牙^[4]^。根据文献[5]报道,异常的出生体重会显著增加成年期肥胖和2型糖尿病等疾病的发生风险。

胎儿期是个体生长发育的关键阶段,这一时期的营养与代谢状况对新生儿的健康和长期发展具有深远的影响^[6]^。新生儿的出生体重与母体在怀孕期间的营养健康状况以及遗传因素紧密相关。因此,评估胎儿在子宫内的代谢状态对出生体重异常机制的理解具有重要意义。传统研究常利用孕妇的脐带血来分析母体代谢对胎儿发育的影响^[7⇓⇓-10]^,但由于胎盘的屏障作用,脐带血可能无法准确反映子宫内的实际环境。同时,胎儿期的生物样本获取也存在挑战。胎便是新生儿在母体分娩后排出的最初粪便,其形成通常始于怀孕的第12周,并在胎儿体内累积至怀孕结束^[11,12]^。胎便由于长时间积累,其代谢物水平较高,能够提供一个较长的检测窗口,被认为是研究母婴健康的理想生物样本^[13,14]^。胎便样本可以在新生儿出生后的前几天内从尿布或尿不湿中收集。胎便中的代谢物主要包括小分子化合物、代谢产物、激素和营养物质,通过对胎便代谢物进行分析,可以了解妊娠期内胎儿在宫内的营养摄取情况、代谢活动和生长发育状态等。例如,胎便中的营养物质水平可以反映胎儿的营养状况,代谢产物水平可以反映胎儿的代谢活动,异常代谢产物的生成表示胎儿可能存在健康问题。

代谢组学通过分析生物样本中的小分子化合物来揭示生物体内的代谢变化^[15]^。将代谢组学技术应用于胎便样本的研究,可以揭示胎儿在母体妊娠期间的代谢变化,从而为探讨胎儿营养摄入、生长发育以及与出生体重相关的代谢路径提供新的见解^[12]^。随着分析技术的进步,特别是液相色谱-高分辨质谱(LC-HRMS)技术的应用,非靶向代谢组学可以实现胎便样本中所有代谢物的高通量检测分析,并已在母婴健康研究中得到了广泛的应用。在早期研究中,我们课题组^[16]^通过对胎便进行代谢组学分析发现,患有糖尿病的孕妇在妊娠期间的氨基酸、嘌呤和脂质代谢通路相对于健康孕妇均发生了明显的变化,这些变化可能对胎儿的生长发育产生不利影响。尽管如此,目前还没有研究探讨过胎便代谢组与新生儿出生体重之间的关联。

针对上述问题,本研究基于已建立的出生队列,采用基于LC-HRMS的非靶向代谢组学技术,采集新生儿胎便的代谢组数据,对胎便代谢物与胎儿出生体重之间的关系进行分析,以期发现可反映胎儿营养状况、代谢活动和生长发育情况的特定代谢产物,为胎儿发育评估提供新的视角。

## 1 实验部分

### 1.1 仪器与试剂

Dionex Ultimate 3000高效液相色谱-Q-Exactive Orbitrap质谱仪、台式高速冷冻离心机、Savant Speedvac离心浓缩仪(美国Thermo Fisher公司);超声波清洗器(昆山舒美超声仪器有限公司);组织匀浆器(美国MP Biomedicals公司);真空冷冻干燥机(北京博医康实验仪器有限公司);超纯水仪(厦门德维科技有限公司)。

甲醇(色谱纯,德国Merck公司);甲酸(色谱纯,美国Thermo Fisher公司)。

### 1.2 人群资料

本研究基于孝感市孝南区妇幼保健院出生队列展开,所有参与者均已签署知情同意书,研究已获得华中科技大学同济医学院伦理委员会的批准(批件号[2021]IEC(A204))。通过问卷调查收集孕妇的社会人口学特征(如年龄、民族、婚姻状况、详细住址、教育水平、收入状况)、身体测量指标(身高、体重)、生活方式(如吸烟、被动吸烟、饮酒和睡眠情况)、孕产史(孕次、产次、流产次数)等信息。在新生儿出生后,研究人员通过查询医院的妇幼健康信息管理系统来获取新生儿的性别、出生体重(g)、胎龄(d)、出生方式(剖宫产或顺产)等信息,并进行记录。本研究共收集了530份胎便样本,在排除缺失问卷信息及重复样本后,最终获得了484份胎便样本的代谢信息。

### 1.3 样品处理

将新生儿初次排出的胎便样本用木片从尿不湿刮取至标记好的离心管中,并储存于-80 ℃冰箱中备用;使用真空冷冻干燥机对样本进行冷冻干燥,并研磨成细粉,随后称取5 mg胎便粉末置于装有锆珠的离心管中,加入1 mL甲醇进行匀浆处理,匀浆2 min后,将样本在超声波低温水浴中提取30 min,在4 ℃、12000 r/min下离心10 min,收集上清液于新的离心管中;向沉淀中再次加入1 mL甲醇,涡旋1 min后,在相同条件下进行二次提取;合并两次提取得到的上清液,并使用离心浓缩仪浓缩至干燥;将干燥的提取物重悬于300 μL的50%甲醇水溶液中,涡旋处理至完全溶解,在4 ℃、12000 r/min下离心15 min;最后,将上清液转移至进样瓶中,待LC-HRMS分析。从每个样本中各吸取50 μL溶液,混合均匀,并将其作为质量控制(QC)样本,以评估仪器稳定性和实验重复性。

### 1.4 分析条件

#### 1.4.1 色谱条件

色谱柱:Waters ACQUITY UPLC HSS T3(100 mm×2.1 mm, 1.8 μm);柱温:45 ℃;流速:0.3 mL/min;进样量:2 μL。流动相A为0.1%甲酸水溶液,流动相B为0.1%甲酸甲醇溶液。梯度洗脱程序:0~1.0 min, 0B; 1.0~4.0 min, 0B~6%B; 4.0~15.0 min, 6%B~50%B; 15.0~21.0 min, 50%B~100%B; 21.0~23.5 min, 100%B~0B; 23.5~25.0 min, 0B。

#### 1.4.2 质谱条件

电离源:加热电喷雾离子源(HESI),正、负离子扫描模式;喷针电压:3.0 kV;毛细管温度:300 ℃;质谱扫描范围:*m/z* 100~1000;二级质谱碰撞能量:25、35和45 eV。

为避免由仪器波动或操作因素所带来的误差,对样本进行随机进样分析。在实验过程中,将QC样本以一定的间隔插入至样本分析序列中。

### 1.5 数据分析

使用Compound Discoverer 3.1软件(美国Thermo Fisher公司)对所获得的原始数据进行峰提取、峰对齐和数据库鉴定。软件参数如下:质量范围100~1000 Da;质量偏差5×10^-6^;保留时间偏差1 min;最小峰强度500000;色谱信噪比10;加和态离子选择[M+H]^+^和[M-H]^-^;数据库选择mzCloud和mzVault。应用随机森林去除系统误差(systematical error removal using random forest, SERRF)方法对预处理后的代谢物离子特征峰数据进行QC校正。基于总峰面积归一化法对代谢物进行定量分析,把峰面积转换为每个代谢物含量占样本中总代谢物含量的比例,随后筛选出QC样本中变异系数小于30%、mzCloud评分高于60且生物功能明确的重要内源性代谢物,以进行后续的统计分析。

本研究采用描述性统计学方法对研究人群的人口学信息进行分析,连续变量采用平均值±标准差来表示,分类资料采用数值(%)表示。使用SIMCA-P软件建立所有样本的主成分分析(principal component analysis, PCA)模型和不同体重组的正交偏最小二乘判别分析(orthogonal partial least squares discriminant analysis, OPLS-DA)模型。所有的统计检验均为双侧检验,当*P*<0.05时,认为该差异具有统计学意义。以OPLS-DA模型中变量投影重要性(variable importance in the projection, VIP)>1、不同出生体重组之间代谢物的变化倍数(fold change, FC)>1.2或<0.83及*P*<0.05的标准筛选出不同组之间的差异胎便代谢物。通过MetaboAnalyst 5.0(https://www.metaboanalyst.ca)对这些差异代谢物进行通路富集分析。

## 2 结果与讨论

### 2.1 基本人口学信息

孕妇和新生儿的基本人口学信息如表1所示。孕妇的平均年龄为26.2岁,其中大部分孕妇的受教育程度为高中及以下(69.6%)。在研究人群(*n*=484)中,初产孕妇占比为46.9%,孕前身体质量指数(body mass index, BMI)在正常范围(18.5≤BMI<24.0)内的孕妇比例为63.4%,超过半数的孕妇(53.5%)居住在农村地区,家庭人均月收入低于3000元的孕妇占比为46.1%。通过剖宫产分娩的孕妇占比高达60.5%,新生儿的平均胎龄和出生体重分别为274.7 d和3333.0 g。在本研究人群中,低出生体重儿(<2500 g)的发病率为3.3%(16例),而巨大儿(>4000 g)的发病率为7.2%(35例),与我国平均水平基本相当^[17]^。

**表1:** 孕妇及新生儿的人口学信息

Characteristic		*n* (%)
Education		
Less than high school		337 (69.6)
High school and above		147 (30.4)
Pre-pregnancy BMI/(kg/m^2^)		
BMI <18.5		110 (22.7)
18.5≤BMI<24.0		307 (63.5)
24.0≤BMI<28.0		51 (10.5)
BMI≥28.0		16 (3.3)
Parity		
1		227 (46.9)
≥2		257 (53.1)
Residence		
Urban		225 (46.5)
Suburb		259 (53.5)
Household income/RMB		
<3000		224 (46.1)
3000-4999		205 (42.5)
≥5000		55 (11.4)
Newborn gender		
Male		236 (48.4)
Female		248 (51.6)
Delivery mode		
Spontaneous		190 (39.5)
Caesarean		294 (60.5)
Maternal age/years (Mean±SD)^*^	26.2±4.2	
Gestational age/d (Mean±SD)^*^	274.7±8.2	
Birth weight/g (Mean±SD)^*^	3333.0±416.9	

*n* (%): number of pregnant women and the corresponding percentage; BMI: body mass index; *: *n*=484.

### 2.2 胎便代谢物的提取与鉴定

由本课题组先前的研究可知,当分别使用超纯水、甲醇、乙腈、甲醇-氯仿(3∶1, v/v)、乙腈-氯仿(3∶1, v/v)等不同溶剂对胎便代谢物进行提取时,甲醇的提取效果最好^[16]^。

因此,本研究继续选用甲醇作为胎便代谢物的提取溶剂。如图1a所示,当使用HSS T3色谱柱进行分离时,胎便样本中的代谢物在25 min内得到了有效分离。在此基础上,采用SIMCA-P软件建立了所有样本的PCA模型,其中QC样本代表所有样本的平均分布。由PCA得分图可知,QC样本紧密聚类在一起(图1b),表明在数据采集过程中不存在明显的批次效应和仪器漂移等情况,保证了实验数据的可靠性和准确性。随后,根据代谢物在QC样本中的变异系数与mzCloud评分筛选出鉴定准确度高且具备明确生物学功能的内源性代谢物,以用于进一步的统计分析,旨在确定不同出生体重组之间的代谢差异。

**图1:**
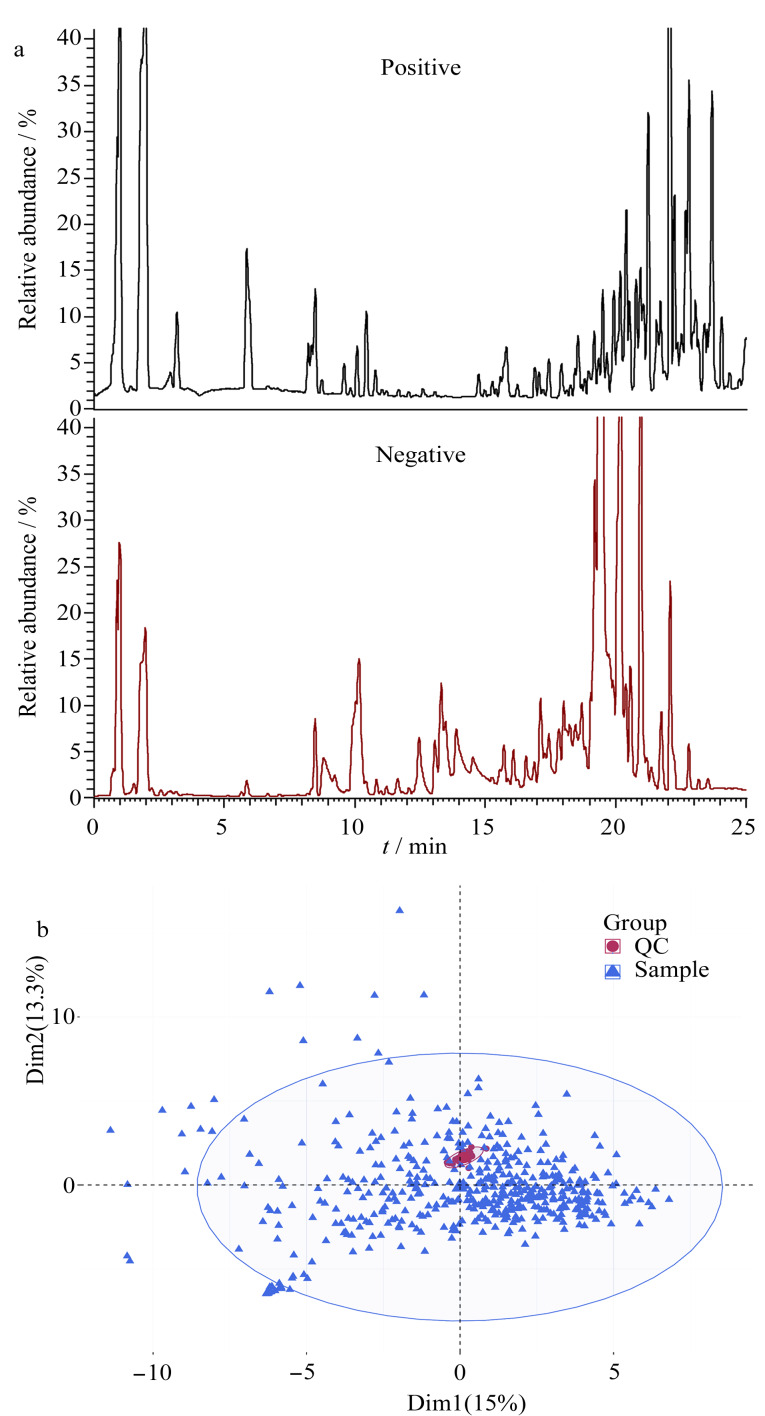
胎便代谢物的(a)色谱图和(b)PCA得分图

#### 2.2.1 胎便代谢组的多变量分析

首先,为了探索不同出生体重组样本之间的差异性,采用PCA模型进行分析。然而,在PCA得分图中未能观察到明显的样本聚类区分,这可能是因为不同出生体重组之间的胎便代谢物水平差异较小,在整体胎便代谢谱中难以观察到显著性变化。为了进一步探究这些差异,采用OPLS-DA模型来区分低出生体重组、巨大儿组和正常体重组的胎便代谢谱。结果如图2所示,无论是低出生体重组还是巨大儿组,均与正常体重组之间产生了明显的区分,并且该模型的稳健性通过999次置换检验得到了验证,说明本方法的聚类分析具有很高的可靠性。

**图2:**
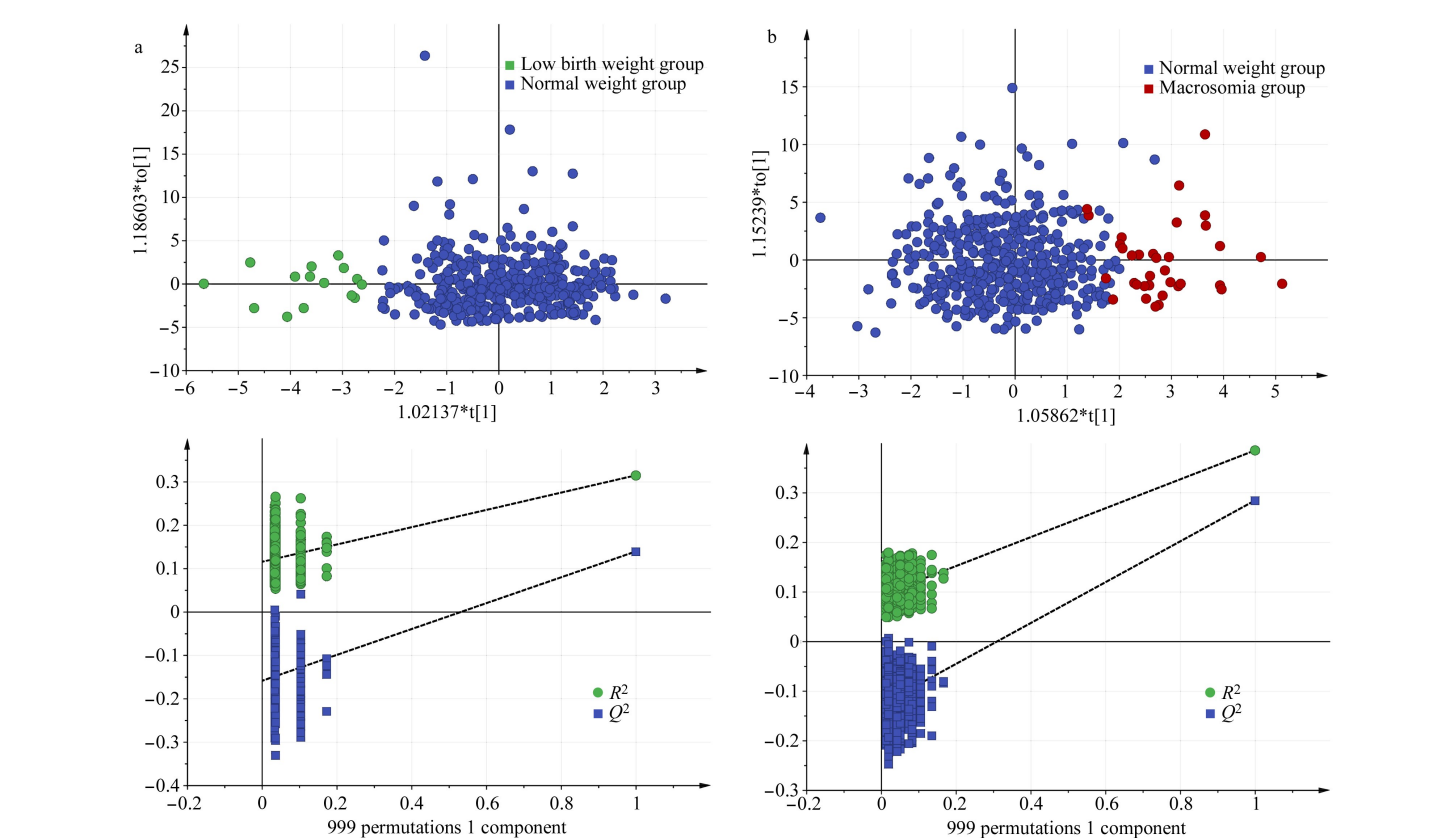
(a)低出生体重组与正常体重组及(b)巨大儿组与正常体重组之间的OPLS-DA分析和置换检验结果

#### 2.2.2 低出生体重儿的差异胎便代谢物

如图3a所示,在低出生体重组与正常体重组之间共筛选到了12个FC符合筛选标准且具有显著差异(*P*<0.05)的胎便代谢物,包括谷氨酸、焦谷氨酸、组氨酸、苏氨酸、谷氨酰胺、胱氨酸、脯氨酸、*N*-乙酰神经氨酸、天冬氨酸、*N*-乙酰-D-半乳糖胺、苯乙酰谷氨酰胺和*β*-亮氨酸。这些代谢物主要为氨基酸及其衍生物,参与氨循环、尿素循环、氨基糖代谢以及丙氨酸和天冬氨酸代谢等关键生物通路(图4)。这些通路的相互作用和调控形成了复杂、精密的分子网络,确保胎儿能够安全有效地利用营养物质,促进其健康发育。

**图3:**
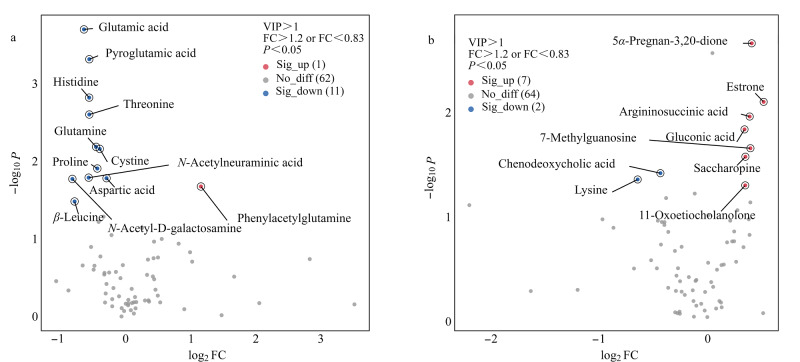
(a)低出生体重组与正常体重组及(b)巨大儿组与正常体重组之间的差异胎便代谢物火山图

**图4:**
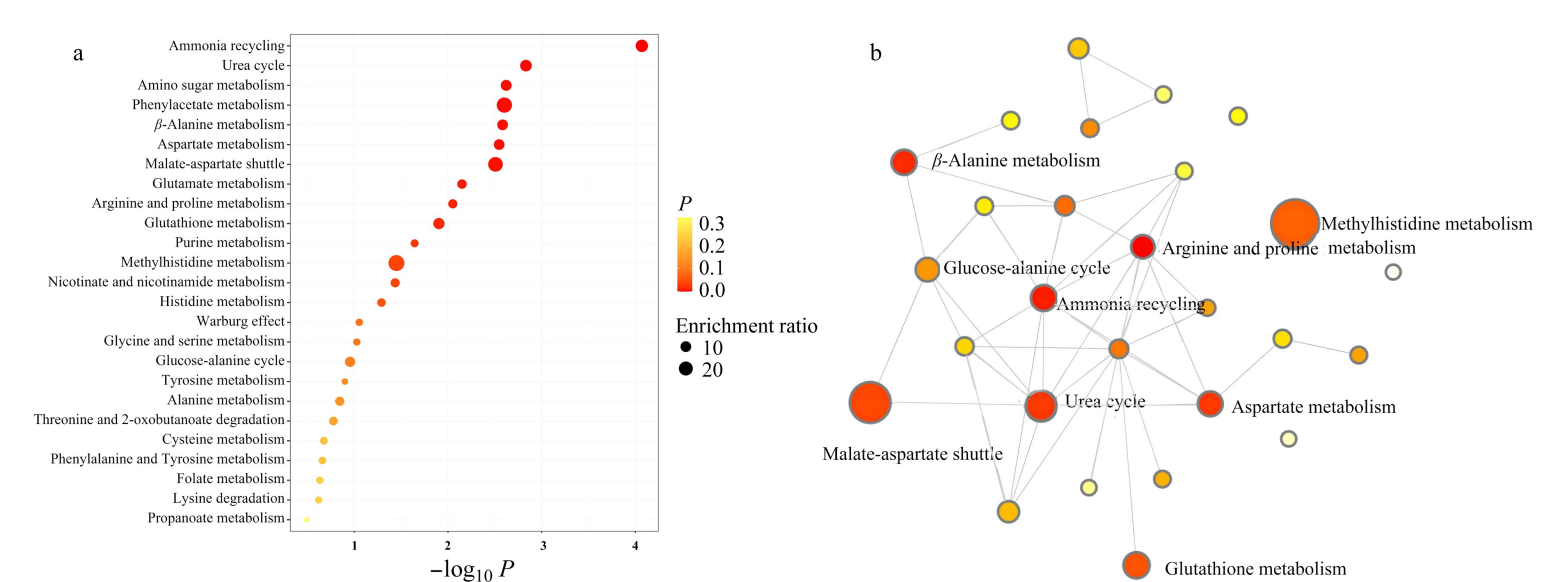
低出生体重组与正常体重组之间差异胎便代谢物的(a)富集分析结果和(b)网络分析结果

在胎儿发育过程中,氨基酸及其衍生物扮演了多重关键角色,不仅参与胎儿肌肉和器官组织的构建,还涉及多条重要代谢途径^[18,19]^。在母体和胎儿体内,这些代谢物的平衡对于胎儿的正常生长和器官发育至关重要。本研究发现在低出生体重组的胎便中,一些关键氨基酸代谢物(如谷氨酸、天冬氨酸、脯氨酸、苏氨酸、亮氨酸和组氨酸)的水平显著低于正常体重组(*P*<0.05),该现象的发生可能与孕妇的饮食、健康状况、胎盘功能以及遗传因素等有关。同时,该发现能够为观察和探索胎儿在宫内的营养状况以及生长发育的潜在影响途径提供重要依据。孕妇的膳食营养不足可能会直接影响上述氨基酸及其衍生物的供应,导致胎盘转运功能异常,从而影响营养物质的传递效率,而遗传因素可能会影响胎儿自身的代谢能力。此外,胎儿体内较低的代谢物水平也可能与胎儿在宫内经历的缺氧、感染或母体所经历的慢性疾病等有关,这些因素可能会激发代谢应激反应,进而破坏胎儿的代谢平衡和生长发育。

在本研究中,低出生体重组与正常体重组之间的谷氨酸水平差异最为显著(*P*<0.01)。作为中心枢纽,谷氨酸不仅是肌肉和神经组织的构建基础,其代谢途径还会直接影响其他氨基酸的合成和利用。谷氨酸可以转变为天冬氨酸,这一过程不仅为三羧酸循环提供了中间产物^[20]^,而且通过天冬氨酸进一步转化生成的瓜氨酸还能够帮助维持氮代谢平衡,并影响胎儿的能量产生和氨解毒过程。先前一项涉及6131名新生儿的研究指出,低出生体重儿(<2500 g)和极端巨大儿(≥5000 g)的发生均与较高水平的谷氨酸有关^[21]^,表明谷氨酸代谢异常会对新生儿的出生体重造成不利影响。低出生体重组中低水平的谷氨酸和天冬氨酸可能意味着胎儿的能量产生和氨解毒过程受损,在某些情况下补充谷氨酸可能会对胎儿的体重有益^[21]^,但天冬氨酸并未显示出相似效果^[19]^。除此之外,谷氨酸也是脯氨酸的前体,而作为胶原蛋白的重要组成部分,脯氨酸对胎儿的结缔组织发育至关重要。胶原蛋白的合成直接关系到胎儿皮肤、骨骼和其他结缔组织的发育,因此谷氨酸的代谢状态不仅影响能量代谢,还决定了胶原蛋白相关组织的发育质量。在本研究中发现,与正常体重组相比,低出生体重组的脯氨酸水平显著降低(*P*<0.05),这可能会导致胎儿在宫内结缔组织发育不全,进而影响新生儿的皮肤、骨骼和其他组织的健康。

作为人体必需氨基酸,苏氨酸和亮氨酸直接影响蛋白质合成和一碳代谢^[22]^。苏氨酸的一碳代谢是关键的DNA合成和修复途径。亮氨酸是支链氨基酸,其对胎儿的肌肉发育和能量产生具有重要作用,同时亮氨酸的代谢产物还能作为能量的直接来源。在本研究中,低出生体重组中低水平的苏氨酸和亮氨酸意味着胎儿的蛋白质合成和能量代谢可能受到干扰,从而导致DNA的合成和修复能力受损,最终影响细胞增殖和器官发育。

作为组织胺的前体,组氨酸在调节胎儿免疫和炎症反应方面发挥着作用^[23]^。组织胺的释放与胎儿免疫系统的成熟程度和其对外界威胁的反应能力密切相关,因此组氨酸的代谢会直接影响胎儿的免疫发展。基于上述问题和本研究结果可推断,低出生体重组中低水平的组氨酸可能会影响胎儿免疫系统的发育,导致免疫功能不全。在本研究中,低出生体重儿胎便代谢物水平的降低可能表明胎儿在宫内面临了营养不足或代谢途径的某些干扰,这些代谢物水平的变化不仅反映了它们在胎儿发育过程中的独立作用,而且体现了代谢网络中各途径之间的相互依赖性与复杂相互作用。

#### 2.2.3 巨大儿的差异胎便代谢物

如图3b所示,在巨大儿组与正常体重组之间共筛选到了7个FC符合筛选标准且具有显著差异(*P*<0.05)的胎便代谢物,包括雌酮、5*α*-孕烷-3,20-酮、11-氧代胆甾醇酮、精氨基琥珀酸、糖胺酮、葡萄糖酸以及7-甲基鸟苷,这些代谢物在巨大儿组中的水平均显著升高。雌酮是一种关键的雌激素,巨大儿胎便中的雌酮水平显著上升表明孕妇体内的雌酮水平偏高。孕期雌酮水平的增长主要由胎盘所调控,其可作为胎盘功能状态的指标。胎盘是胎儿与母体进行营养物和废物交换的关键介质,雌酮水平升高可能意味着胎盘功能的加强,从而有利于将更多营养物质传递到胎儿体内^[24]^。另一方面,孕妇体内雌激素水平的升高可能会使胰岛素敏感性发生改变,进而在孕晚期引起孕妇体内胰岛素抗性增强,促使更多的胰岛素和葡萄糖等营养物质通过胎盘传递给胎儿,特别是葡萄糖,这可能会刺激胎儿胰腺产生更多的胰岛素,从而加快胎儿的生长速度。此外,较高的雌激素水平也可能促进脂肪组织的发育和脂肪酸的合成,导致胎儿体内的脂肪积聚加剧,引起体重增长。巨大儿组中5*α*-孕烷-3,20-酮和11-氧代胆甾醇酮水平的显著上升表明,与胎儿生长和能量代谢相关的激素调节可能存在异常,暗示了5*α*-孕烷-3,20-酮和11-氧代胆甾醇酮在胎儿体内脂肪累积及体重增加过程中的潜在作用。以上这些差异胎便代谢物可能会共同导致巨大儿的产生。

精氨基琥珀酸水平的改变与尿素循环及氨基酸代谢有关,它们可能影响胎儿的生长速率;巨大儿组中糖胺酮及葡萄糖酸的水平差异可能指示糖代谢途径的变化,这与巨大儿的糖耐量异常和糖尿病风险提高有关。7-甲基鸟苷的水平变化涉及RNA修饰和核苷代谢通路,其在体内的水平对基因表达调控和表观遗传学特征都具有重要意义。这些代谢物自身的分子功能改变和分子间的相互作用可能直接或间接影响胎儿的能量平衡、细胞增殖、组织发育以及激素反应等多个功能发育过程,针对这些代谢物进行研究有助于为胎儿的过度生长提供代谢组学基础。

## 3 结论

本研究利用LC-HRMS对不同出生体重新生儿的胎便代谢组进行了系统分析。结果发现,低出生体重组与正常体重组以及巨大儿组与正常体重组之间的代谢物水平差异具有明显的不同,这些差异可能源于胎儿发育过程中多种生物学与环境因素的交互作用。首先,胎儿生长受限及胎儿过度生长的病理、生理机制截然不同,前者可能与胎盘功能障碍、母体营养不足或氧气供应缺陷有关,而后者可能与母体代谢性疾病或胎盘激素过量有关;其次,内分泌系统的差异调节可能导致激素及相关代谢物的表达水平存在显著差异,这反映了胎儿生长调控的分子层面异质性;此外,环境因素(如母体的生活方式、饮食习惯和健康状况)以及外界环境的毒素、药物和感染暴露等,也可能会影响胎儿的代谢状态。本实验的研究结果可能反映了胎儿在生长发育过程中的不同代谢途径与调控机制,为未来胎儿发育相关疾病的深入研究提供了潜在代谢标志物和研究方向。

同时,本研究也存在一定的局限性。研究中可能存在未考虑的潜在混杂因素,如孕妇的饮食习惯、生活方式等,这些因素可能对代谢物的检测和分析产生影响,未来的研究需要更加全面地考虑这些因素对研究结果的潜在影响。此外,本研究仅基于孝感市出生队列开展,局限于特定的地区和人群,未来建立多中心、多种族、多地区的研究方法将有助于验证研究结果的普适性。
